# Supplementary material for: The ALGOVUE Clinical Trial: Effects of the Daily Consumption of Eggs Enriched with Lutein and Docosahexaenoic Acid on Plasma Composition and Macular Pigment Optical Density
Source: Nutrients. 2021 Sep 24;13(10):3347. doi: 10.3390/nu13103347 (PMC8541434; doi:10.3390/nu13103347)
Supplement: Supplementary file 1 [file nutrients-13-03347-s001.zip › nutrients-1332365-supplementary.pdf]

Supplemental Table S1: Baseline food-intake of the participants

Baseline Food-Intake	Standard Group Mean (SD)	Enriched Group Mean (SD)	p-Value
Total Energy (kcal)	2224.5 (548.9)	2025.6 (587.7)	0.09
Proteins (g)	85.6 (26.4)	79.7 (20.9)	0.23
Carbohydrates (g)	231.2 (64.1)	213.6 (75.3)	0.22
Sugar (g)	74.1 (28.5)	72.3 (30.1)	0.76
Total fats (g)	90.6 (29.7)	80.9 (28.7)	0.11
Saturated fatty acids (g)	27.1 (10.4)	25.3 (9.2)	0.35
Monounsaturated fatty acids (g)	22.6 (8.6)	22.3 (8.6)	0.87
Polyunsaturated fatty acids (g)	9.9 (6.3)	9.1 (4.5)	0.46
DHA (g)	0.018 (0.04)	0.015 (0.04)	0.72
Total cholesterol (mg)	301.9 (147.5)	297.1 (121.9)	0.86
Fibers (g)	18.8 (23.2)	15.6 (6.2)	0.35
Carotenoids			
Lutein + Zeaxanthin (µg)	275.4 (257.5)	384.4 (486.7)	0.18
β-carotène (µg)	2013.2 (1511.8)	2060.6 (1783.4)	0.89
Vitamins			
Vitamin A (µg)	356.8 (421.7)	367.6 (352.9)	0.89
Vitamin E (mg)	7.3 (3.4)	7.5 (3.6)	0.76
Vitamin D (µg)	2.2 (1.6)	1.7 (1.2)	0.11
Sodium (mg)	2170.7 (820.3)	2024.9 (746.2)	0.36
Calcium (mg)	676.1 (299.4)	640.1 (262.9)	0.54
Magnesium (mg)	235.8 (94.2)	218.9 (70.7)	0.33
Potassium (mg)	2479.9 (946.7)	2376.0 (657.9)	0.54
Iron (mg)	0.104 (0.310)	0.099 (0.221)	0.92
Selenium (µg)	41.2 (21.1)	36.4 (13.3)	0.18

Abbreviations: SD= standard deviation, DHA= docosahexaenoic acid.
